# Cystatin-B Negatively Regulates the Malignant Characteristics of Oral Squamous Cell Carcinoma Possibly *Via* the Epithelium Proliferation/Differentiation Program

**DOI:** 10.3389/fonc.2021.707066

**Published:** 2021-08-24

**Authors:** Tian-Tian Xu, Xiao-Wen Zeng, Xin-Hong Wang, Lu-Xi Yang, Gang Luo, Ting Yu

**Affiliations:** ^1^Department of Periodontics, Affiliated Stomatology Hospital of Guangzhou Medical University, Guangzhou Key Laboratory of Basic and Applied Research of Oral Regenerative Medicine, Guangzhou, China; ^2^Department of Oral Pathology and Medicine, Affiliated Stomatology Hospital of Guangzhou Medical University, Guangzhou Key Laboratory of Basic and Applied Research of Oral Regenerative Medicine, Guangzhou, China

**Keywords:** oral squamous cell carcinoma, head and neck squamous cell carcinoma, cysteine cathepsin, cystatin-B, epithelial proliferation/differentiation, WGCNA, GSEA

## Abstract

Disturbance in the proteolytic process is one of the malignant signs of tumors. Proteolysis is highly orchestrated by cysteine cathepsin and its inhibitors. Cystatin-B (CSTB) is a general cysteine cathepsin inhibitor that prevents cysteine cathepsin from leaking from lysosomes and causing inappropriate proteolysis. Our study found that CSTB was downregulated in both oral squamous cell carcinoma (OSCC) tissues and cells compared with normal controls. Immunohistochemical analysis showed that CSTB was mainly distributed in the epithelial structure of OSCC tissues, and its expression intensity was related to the grade classification. A correlation analysis between CSTB and clinical prognosis was performed using gene expression data and clinical information acquired from The Cancer Genome Atlas (TCGA) database. Patients with lower expression levels of CSTB had shorter disease-free survival times and poorer clinicopathological features (e.g., lymph node metastases, perineural invasion, low degree of differentiation, and advanced tumor stage). OSCC cell models overexpressing CSTB were constructed to assess the effects of CSTB on malignant biological behaviors and upregulation of CSTB inhibited cell proliferation, migration, and invasion *in vitro*. Weighted gene correlation network analysis (WGCNA) and gene set enrichment analysis (GSEA) were performed based on the TCGA data to explore potential mechanisms, and CSTB appeared to correlate with squamous epithelial proliferation-differentiation processes, such as epidermal cell differentiation and keratinization. Moreover, in WGCNA, the gene module most associated with CSTB expression (i.e., the brown module) was also the one most associated with grade classification. Upregulation of CSTB promoted the expression levels of markers (LOR, IVL, KRT5/14, and KRT1/10), reflecting a tendency for differentiation and keratinization *in vitro*. Gene expression profile data of the overexpressed CSTB cell line were obtained by RNA sequencing (RNA-seq) technology. By comparing the GSEA enrichment results of RNA-seq data (from the OSCC models overexpressing CSTB) and existing public database data, three gene sets (i.e., apical junction, G2/M checkpoint, etc.) and six pathways (e.g., NOTCH signaling pathway, glycosaminoglycan degradation, mismatch repair, etc.) were enriched in the data from both sources. Overall, our study shows that CSTB is downregulated in OSCC and might regulate the malignant characteristics of OSCC *via* the epithelial proliferation/differentiation program.

## Introduction

As the sixth most common cancer in the world, head and neck squamous cell carcinoma (HNSCC) continues to rise yearly, and oral squamous cell carcinoma (OSCC) accounts for 90% of HNSCC cases in the region (1–4). The survival of OSCC patients, especially those with distant metastases, remains low, at approximately 39% (1, 4, 5). Moreover, due to the special anatomical location of the oral cavity, OSCC patients often experience great psychological stress and compromised quality of life (6). To date, the potential pathogenesis, including the progression mechanism, of OSCC is not fully understood. It is generally accepted that the initiation and development of OSCC is a complex process requiring the accumulation of genomic alterations, which is modified by individual genetic predisposition and environmental carcinogenic risk factors (7–9). It is necessary and challenging for clinical oncology and precision medicine science to understand the carcinogenic and progression mechanisms of OSCC, to identify biomarkers for early screening and to establish an accurate prognostic evaluation system, all of which would contribute to the reduction in the incidence and improvement of both the survival rate and living quality of tumor-bearing patients (10–12).

Proteolysis has a vital role in the normal life activities of organisms. Disorders between the enzymatic reaction and the inhibitory reaction of the proteolytic cascade process are one of the malignant characteristics of tumors, and they also lead to tumor invasion and metastasis (13–16). Numerous evidences have indicated that cysteine cathepsins play a crucial role in these processes (17–20). Cysteine cathepsin can degrade the proteins of the extracellular matrix (ECM) and reshape the tumor microenvironment by regulating a variety of cytokines and thus participate in tumor growth, invasion, angiogenesis, and metastasis (21). Compared with the fully elucidated role of the cysteine protease family in tumors, research on its inhibitors in tumors is relatively limited. Most studies only focus on certain types of inhibitors and are not in-depth enough. As an inhibitor of cysteine cathepsin, cystatin-B (coded by *Cstb* gene) is considered a general cysteine cathepsin inhibitor in mammalian cells, preventing cysteine cathepsin from leaking from lysosomes and causing inappropriate proteolysis (18, 22–24). CSTB plays a bimodal role in cancer. Recent studies have associated CSTB with various cancers [e.g., hepatocellular carcinoma (HCC) (25–28), epithelial ovarian tumors (29, 30), breast cancer (31), laryngeal squamous cell carcinoma, and esophageal squamous cell carcinoma, and esophageal squamous cell carcinoma (ESCC) (32, 33)], in which the expression, in which the expression of CSTB is changed in different directions. For instance, CSTB was increased in HCC (25–28), epithelial ovarian tumors (29, 30), and breast cancer (31), while decreased in laryngeal squamous cell carcinoma and ESCC (32, 33). Some studies have indicated that increased CSTB is related to a poorer prognosis in bladder cancer (34) and a higher risk of tumor metastasis in HCC (27). However, most of the existing studies on the correlation between CSTB and tumors just provide observational evidence and whether CSTB has a causal role in cancers (including OSCC) is unclear. Only a few studies found that CSTB may participate in tumors by regulating cell apoptosis and oxidative stress (31). Only one noninterventional study based on human OSCC tissue specimens reported that low expression of CSTB in the invasive tumor front was correlated with local tumor recurrence. In addition, CSTB-specific peptides in saliva were associated with lymph node metastasis (35). However, the single study reached a conclusion based only on OSCC specimens but lacked a nontumor (normal control) group. Moreover, there is a lack of in-depth and comprehensive mechanistic studies on the role of CSTB in the progression of OSCC. Based on the above evidence, we hypothesized that CSTB may play a role in OSCC. Our research aimed to identify the role of CSTB in OSCC and to explore the relevant mechanisms.

## Materials And Methods

### Cell Line Acquisition and Culture

The human tongue squamous cell carcinoma cell lines SCC9 and SCC25 (ATCC, ATCC^®^ CRL-1629™ and CRL-1628™, Manassas, VA, USA) and the normal oral epithelial keratinocyte line HOK (AULU, Guangdong, China) were purchased and cultured in Dulbecco’s modified Eagle’s medium (DMEM)/F12 (Gibco, Waltham, MA, USA) and DMEM, respectively. The complete cell culture medium contained 10% fetal bovine serum (FBS, Gibco) and 1% penicillin/streptomycin (Gibco), while the medium for carcinoma cells was supplemented with an additional 400-ng/ml hydrocortisone (MedChemExpress, Monmouth Junction, NJ, USA). The serum-free cell culture medium for carcinoma cells was prepared as DMEM/F12 containing 1% penicillin/streptomycin and 400 ng/ml hydrocortisone for a number of subsequent experiments (e.g., enzyme-linked immunosorbent assay, wound-healing assay, cell migration, and invasion assays). The cells were cultured in a 37°C, 5% CO_2_ incubator.

### Clinical Patient Specimen Collection and Immunohistochemistry Staining for Cystatin-B

The studies involving human participants were approved by the Ethics Committee of the Stomatology Hospital of Guangzhou Medical University. Twenty-three primary OSCC specimens and fifteen normal oral tissues were obtained from the Department of Periodontics and Oral Mucosal Diseases, Stomatology Hospital of Guangzhou Medical University. All OSCC specimens were from samples pathologically diagnosed as OSCC. Normal specimens were excess tissues that needed to be removed due to tooth extractions or oral surgeries, which were also examined as normal oral mucosal epithelium by pathology. The pathological grading (36) of tissue sections was classified blindly by a single pathologist (Xin-Hong Wang). Immunohistochemistry (IHC) staining for cystatin-B was performed in the above tissues (*Materials and Methods S1.1*). After staining, five fields of view were randomly selected to take images under an upright optical microscope. The integrated optical density (IOD) value of each point on the image was collected by ImageJ and then the average density (%, IOD/target distribution area) was calculated, representing the expression level of CSTB in certain specimens. Immunostaining was analyzed by a researcher who was blinded to the pathological grade of the samples. Correlation analysis between CSTB expression and the degree of pathological differentiation was conducted.

### Upregulation of CSTB in OSCC Cell Lines by Lentiviral Transfection

The commercial recombinant lentivirus (OBIO, Shanghai, China) named lenti-CSTB was utilized to overexpress CSTB, and an empty carrier lentivirus named lenti-NC was used as a negative control. SCC9 and SCC25 cells were infected with the above lentivirus (MOI = 30) and screened with puromycin (3 µg/ml) for 15 days. Quantitative reverse transcription-polymerase chain reaction (qRT-PCR) and Western blotting were used to validate the overexpression of CSTB at the mRNA and protein levels, respectively. After successful transfection, the cell lines (i.e., SCC9/25-lenti-CSTB and SCC9/25-lenti-NC) were cultured with complete medium containing puromycin (1 µg/ml) for subsequent analysis.

### RNA Isolation and Quantitative Real-Time PCR

Total RNA was extracted from the cells using an RNA extraction kit (AG21017, Accurate Biology, Hunan, China). Extracted RNA was analyzed for quantity and quality by measuring A260/A280 with a spectrophotometer (NanoDrop 2000, Thermo Fisher Scientific, Waltham, MA, USA). RNA integrity was confirmed by 1.5% agarose gel electrophoresis. For qRT-PCR, 1 μg of total RNA was used to synthesize cDNA (AG11706, Accurate Biology, China). qRT-PCR was performed using a SYBR Green qPCR kit (AG11718, Accurate Biology, China). Relative mRNA expression was normalized to that of the internal *GAPDH* control. The primer sequences used are listed in the **Table S1**. The relative expression of targeted genes was calculated using the 2^−△△Ct^ method (37). Each test was repeated at least three times.

### Western Blotting Analysis

Total protein was isolated from cell samples using precooled cell lysis buffer (Cell Signaling Technology, Danvers, MA, USA) with protease inhibitor (RayBiotech, Guangzhou, China) and quantified using a BCA protein assay kit (Beyotime, Shanghai, China). Equal protein extracts (20 µg) were separated by SDS-PAGE and transferred to polyvinylidene fluoride membranes (Millipore, Burlington, MA, USA). After successive incubations with the primary antibody and the secondary antibody, the target protein was visualized by chemiluminescence using an ECL kit (Beyotime). The following antibodies were used in the Western blot assay: anti-loricrin polyclonal antibody (1:1,000, 55439-1-AF, Proteintech, Wuhan, China), anti-involucrin polyclonal antibody (1:1,000, 55328-1-AP, Proteintech), anti-cystatin-B monoclonal antibody (1:1,000, 66712-1-Ig, Proteintech), anti-beta-actin monoclonal antibody (1:10,000, 66009-1-Ig, Proteintech), anti-GAPDH antibody (1:10,000, EPR16891, Abcam, Cambridge, UK), goat anti-mouse IgG (H+L) antibody (HRP) (1:10,000, SA00001-1, Proteintech), and goat anti-rabbit IgG H&L (HRP) antibody (1:10,000, ab205718, Abcam). GAPDH or beta-actin served as the internal controls to calculate the relative expression of the targeted proteins.

### Enzyme-Linked Immunosorbent Assay

Cells were inoculated at a density of 1 × 10^6^ in complete medium, which was replaced by serum-free medium after 2 days of culture. After another 2 days, the supernatant was collected, centrifuged (4°C, 2,000 rpm, 10 min), and stored at −80°C. The protein levels of CSTB in cell supernatants were examined by an enzyme-linked immunosorbent assay (ELISA) kit (R&D Systems, DCYB00) according to the manufacturer’s instructions. Each test was repeated at least three times.

### Cell Proliferation Assay

Transfected SCC9 and SCC25 cells were inoculated with complete medium in 96-well plates at a density of 3,000 cells per well. Cell proliferation was detected using a Cell Counting Kit-8 (Dojindo, Kumamoto, Japan) at 1, 2, 3, and 5 days according to the manufacturer’s instructions. Each test was repeated at least three times.

### Colony Formation Assay

Three thousand transfected SCC9 cells and SCC25 cells were seeded respectively in six-well plates and cultured for 10 days in complete medium. Afterward, the colonies were fixed with 4% paraformaldehyde and stained with crystal violet. The colonies that contained more than 50 cells were counted. Each test was repeated at least three times.

### Wound-Healing Assay

For the wound-healing assay, transfected SCC9 and SCC25 cells were first incubated and cultured with complete medium. A scratch was made with a sterile pipette tip after a confluent monolayer of cells was formed. Afterward, the cells were washed with phosphate-buffered saline (PBS) and cultured with serum-free medium. Images were taken at 0, 24, and 36 h postwounding. The wound-healing areas were assessed by ImageJ to calculate the wound-healing rate. Wound-healing rate% = [Area*_t_*_0_ − Area*_t_*_1_]/Area*_t_*_0_ × 100% (Area*_t_*_0_ is the area of the wound measured immediately after scratching, and Area_t1_ is the area of the wound measured *t*1 hours after scratching). In our research, the area of the wound was measured at 0, 24, and 36 h. Each test was repeated at least three times.

### Cell Migration and Invasion Assays

Cells were seeded at a density of 1 × 10^5^ (a density of 5 × 10^4^ for transfected SCC9 cells for the migration assay) in serum-free DMEM/F12 medium in the upper wells of Transwell chambers (8 μm pore size, Corning, New York, NY, USA), while the lower wells were filled with complete medium containing 10% FBS. Chambers for the invasion assay were coated with 100 µl of Matrigel (200 μg/µl, Corning) and incubated for 2 h at 37°C. Cells in the upper layer were removed with a swab after 24 h of culture, and cells on the bottom membrane were fixed with 4% paraformaldehyde and stained with crystal violet. The results of each group were photographed at five randomized visual fields, and the experiments were repeated three times.

### Bioinformatics Analysis for Identifying CSTB Expression

Five expression microarray series containing OSCC tumor and normal samples were downloaded from the Gene Expression Omnibus (GEO) database (https://www.ncbi.nlm.nih.gov/geo/) (**Table S2**). One expression microarray series containing OSCC tumor and oral leucoplakia samples was also downloaded from GEO (**Table S2**). The Cancer Genome Atlas (TCGA) OSCC mRNA normalized count data and clinicopathological information of 329 OSCC tissues and 32 matched normal oral mucosal epithelial tissues were downloaded from the Genomic Data Commons Data Portal (https://cancergenome.nih.gov/). The *Cstb* expression levels of both the OSCC and control groups in all datasets were extracted and compared using R Studio software.

### Survival Analysis and Clinicopathological Correlation Analysis

TCGA patients with clinicopathological information were used for clinicopathological correlation analysis. Among the patients, 291 OSCC patients with complete clinicopathological and survival data were selected for survival analysis. Next, these 291 patients were divided into high- and low-expression groups based on the median expression level of *Cstb* for weighted gene correlation network analysis (WGCNA) and gene set enrichment analysis (GSEA).

### Weighted Gene Coexpression Network Analysis

The gene expression data and sample clinical information were downloaded from TCGA. First, variance analysis of 22,862 protein-coding genes (PCGs) was performed, the top 25% (5,716 genes) of which were selected for WGCNA. The WGCNA package in R Studio software was applied to construct a gene coexpression network. The soft threshold power (*β* = 6) was selected to ensure a scale-free network. The adjacency matrix was transformed into a topological overlap matrix (ToM), and the corresponding dissimilarity was calculated. The module eigengenes were calculated to identify modules that were significantly associated with the clinical feature information. In this process, the mRNA expression level of *Cstb* was also regarded as a feature and was inputted into the correlation analysis, aiming to identify the functional modules related to its expression. Modules with a high correlation coefficient were considered candidate modules related to clinical features and were selected for subsequent analysis. Finally, Gene Ontology (GO) and Kyoto Encyclopedia of Genes and Genomes (KEGG) enrichment analyses were performed to reveal the functions of the genes in the target modules. *p-*Value ≤0.05 and *q*-value ≤0.05 were considered statistically significant. The detailed method for WGCNA is shown in the *Materials and Methods S1.2*.

### mRNA Sequencing of the Transfected Cell Line

The total RNA of transfected SCC25 cells (lenti-CSTB/NC-SCC25) was extracted for further RNA-seq analysis (*n* = 3). High-throughput sequencing was conducted by SEQHEALTH (Wuhan, China). The raw reads of samples were obtained. The clean reads were mapped to the reference genome of Homo sapiens (Homo_sapiens, GRCh38) using STRA software (version 2.5.3a) with default parameters. The reads mapped to the exon regions of each gene were counted by FeatureCounts (Subread-1.5.1; Bioconductor), and then the reads per kilobase of exon model per million mapped reads (RPKM) were calculated. Genes differentially expressed between groups were identified using the edgeR package. A *p*-value cutoff of 0.05 and fold-change cutoff of 1.5 were used to judge the statistical significance of gene expression differences. Afterwards, the differentially expressed genes (DEGs) were compared with the mutational cancer driver gene set published in IntOGen (https://www.intogen.org/search#driver-genes:table) (38). Detailed methods for mRNA sequencing are shown in the *Materials and Methods S1.3*. Details of the mutational cancer driver genes are shown in the **Supplementary Data Sheet 1**.

### Gene Set Enrichment Analysis

First, samples obtained from TCGA were divided into high- and low-expression groups based on the median expression level of *Cstb*. A similar analysis was also performed based on the RNA-seq data acquired from *mRNA Sequencing of the Transfected Cell Line*. KEGG and GO analyses were used to explore potential cancer-related biological pathways, while Hallmark GSEA was used to correlate the expression level of *Cstb* with the biological functions of oncogenes. *p*-Value ≤0.05 and FDR ≤25% were considered statistically significant.

### Statistical Analysis

The data were analyzed using data statistics software (GraphPad Prism 7.0 and SPSS Statistics 16.0). The data are presented as the means and standard deviations. Student’s *t*-test was performed for two independent samples, while analysis of variance (ANOVA) was used for multiple independent samples, and *post-hoc* comparisons were made. The correlation between CSTB expression and the degree of pathological differentiation in IHC analysis was determined using Fisher’s exact test. Logistic regression analysis was performed to analyze the relationship between the expression of CSTB and the clinicopathological characteristics of OSCC patients. The correlation between genes in WGCNA was analyzed using Pearson’s correlation. *p*-Value <0.05 was considered statistically significant.

**Figure 1:**
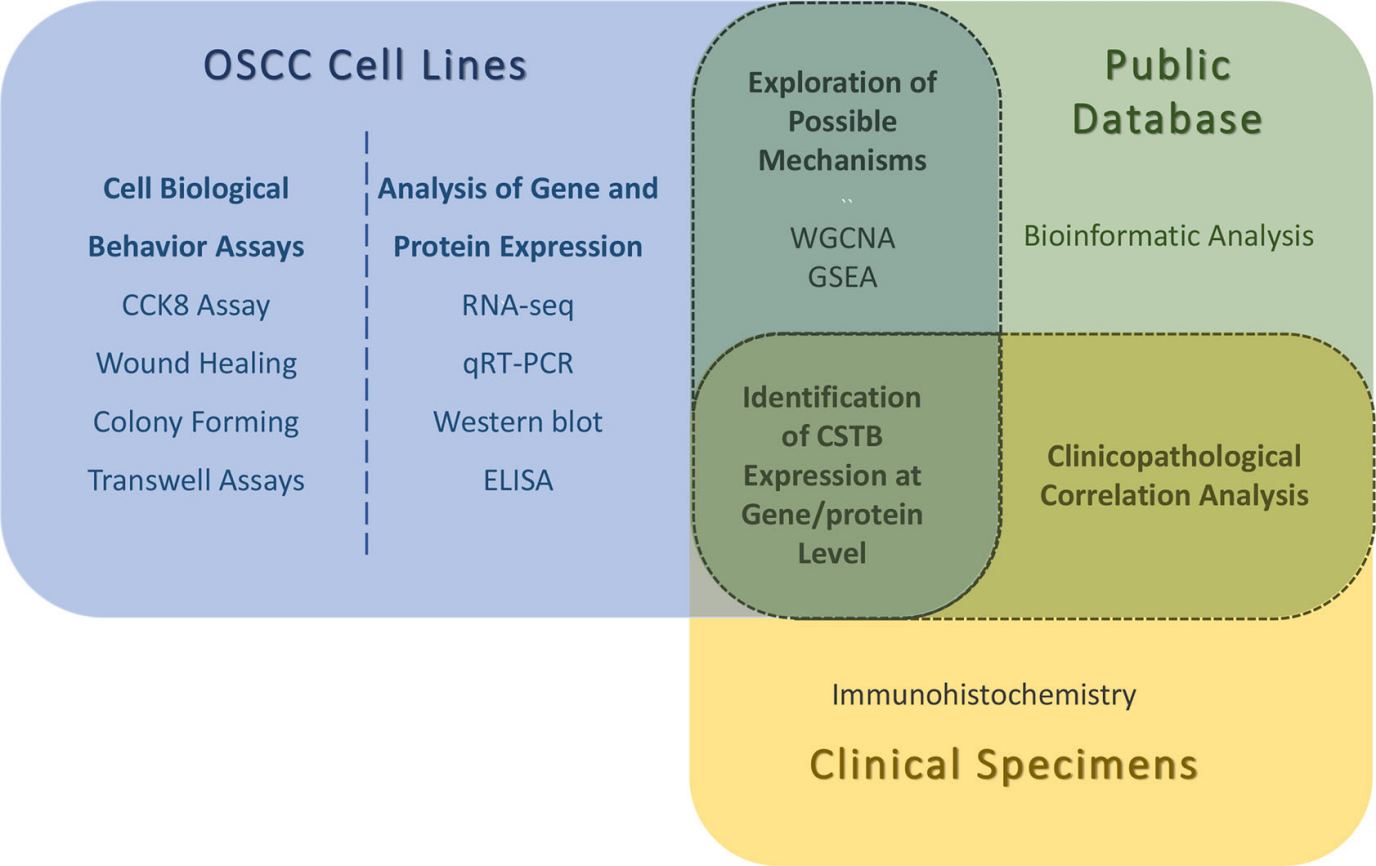
Snapshot of the entire experimental process. WGCNA, weighted gene correlation network analysis; GSEA, gene set enrichment analysis. The identifications of CSTB expression at gene/protein levels are obtained by *in vitro* (OSCC cell lines) and *in vivo* (public database/clinical specimens) experiments. Possible mechanisms for the involvement of CSTB in OSCC were explored using OSCC cell lines and public database data together. Using OSCC tissue samples (from public databases and independent clinical specimens) for clinicopathological correlation analysis.

A snapshot of the entire experimental process is shown in **Figure 1**.

## Results

### CSTB Is Downregulated in OSCC Both *In Vivo* and *In Vitro*

**Figure 2:**
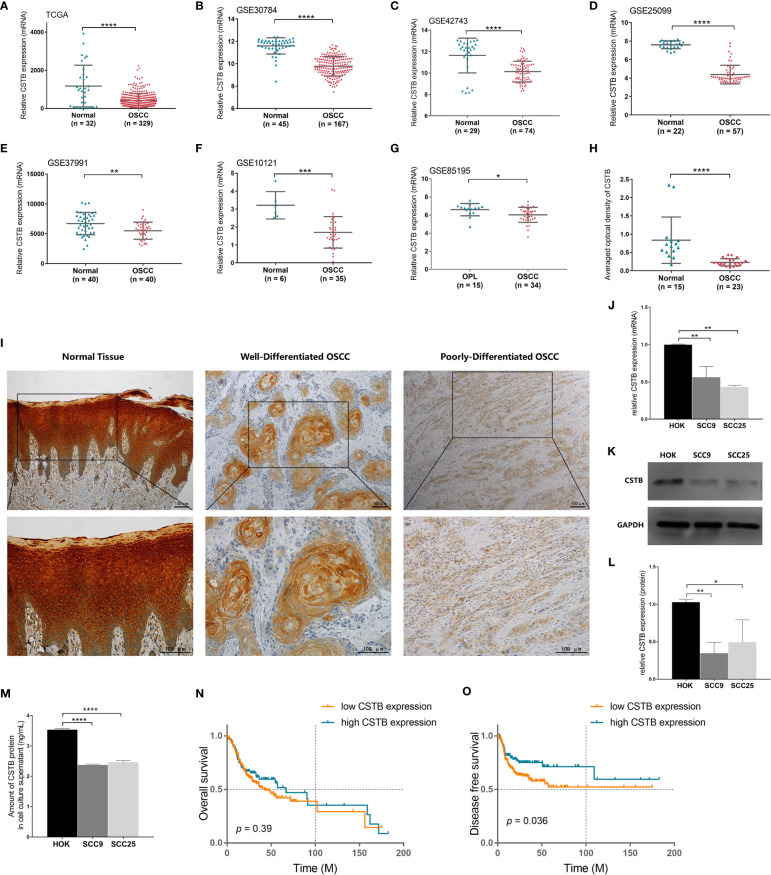
CSTB is downregulated in OSCC and is associated with poor prognosis. Relative expression of CSTB mRNA in OSCC tissues (*n* = 329) and matched normal tissues (*n* = 32) from the TCGA database. **(B**–**F)** Relative expression of CSTB mRNA in OSCC tissues and normal tissues from the GEO database. The sample size of each group is shown in the figures. **(G)** Relative expression of CSTB mRNA in OSCC tissues (*n* = 34) and oral leucoplakia (OPL) tissues (*n* = 15) from the GEO database. **(H)** Average optical density of CSTB in normal (*n* = 15) and OSCC (*n* = 23) tissues in IHC analysis. **(I)** IHC staining of CSTB in normal and OSCC tissues. Magnification at ×100 (upper panel) and ×200 (lower panel); scale bars, 200 μm. **(J)** The relative expression of CSTB mRNA in the oral epithelial keratinocyte line and OSCC cell lines was quantified by RT-qPCR. **(K, L)** CSTB protein expression in the oral epithelial keratinocyte line and OSCC cell lines was identified by Western blot analysis **(K)** and quantitatively analyzed **(L)**. **(M)** The content of CSTB in the culture supernatant in the oral epithelial keratinocyte line and OSCC cell lines was quantified by ELISA. **(N, O)** Kaplan-Meier survival curves of overall survival and disease-free survival in the high and low CSTB expression groups. Error bars represent the standard deviation. *p < 0.05, **p < 0.01, ***p < 0.001, ****p < 0.0001.

Based on the data from public databases, the expression of *Cstb* was lower in OSCC tissues than in normal oral mucosal epithelial tissues (**Figures 2A–F**) and potentially malignant disorder tissues (i.e., oral leucoplakia tissues, **Figure 2G**). IHC analysis of tissue samples (independently collected from OSCC patients by the coauthors) also showed a downregulation of protein CSTB expression in OSCC tissues (**Figure 2H**). CSTB was located in the epithelial structure of OSCC tissues, similar to the distribution of CSTB in the epithelial layer of normal oral mucosal epithelial tissues. The staining of CSTB was lighter in OSCC and showed preferential localizations in well-differentiated structures (i.e., cancer nests and keratinized pearls). There was little staining of CSTB in either the tumor stroma in OSCC or subepithelial tissues in control tissues (**Figure 2I**). Correlation analysis revealed that the levels of CSTB were positively correlated with the degree of tissue differentiation (*p* < 0.05, **Table 1**). For subcellular localization, positive CSTB staining was mainly distributed in the cytoplasm and occasionally distributed in the nucleus in both OSCC and control tissues (**Figure 2I**).

**Table 1:** Association between CSTB expression and tissue differentiation.

Degree of differentiation^a^	Low CSTB expression (*n*)^b^	High CSTB expression (*n*)^b^	Sum	*p*-value^c^
Well	5	10	15	0.027
Moderate to poor	7	1	8	
Sum	12	11	23	

a Grade 1 was regarded as well differentiation. Grades 2 and 3 were regarded as moderate ~ poor differentiation.

b Patients were divided into two groups (low and high CSTB expression) based on the median expression of CSTB.

c Statistical method: Fisher’s exact test; statistical significance at p < 0.05.

The results of the *in vitro* experiments were consistent with those of the *in vivo* experiments. CSTB was downregulated intracellularly in common OSCC cell lines (i.e., SCC9 and SCC25) compared with the normal cell line (i.e., HOK) (**Figures 2J–L**). Interestingly, extracellular CSTB (i.e., in the cell culture supernatant) was also downregulated in OSCC cell lines (**Figure 2M**).

### Downregulation of CSTB in Tissues Is Associated With Poor Prognosis in OSCC Patients

To explore whether there was a link between CSTB expression and the clinical prognosis of OSCC patients, a correlation analysis between them was conducted based on the data extracted from the TCGA database. There was no significant difference in the overall survival rates between the two groups (**Figure 2N**). However, the disease-free survival (DFS) rate (an indirect indicator of cancer recurrence and metastasis) of the low *Cstb* expression group was lower than that of the high *Cstb* expression group (**Figure 2O**). Logistic regression analysis indicated that patients with lower expression levels of *Cstb* had poorer clinicopathological features (e.g., lymph node metastases, perineural invasion, low degree of differentiation, and advanced tumor stage). Patients with lymphovascular invasion tended to have lower *Cstb* expression (*p* = 0.06). No association was found between the abundance of *Cstb* and demographic characteristics, including age and sex (**Table 2**).

**Table 2:** Association between CSTB expression and clinicopathological features.

Clinicopathological feature	Total (*n*)	Odds ratio for CSTB expression	*p*-Value
Age (continuous)	329	1.00 (0.98~1.02)	0.98
Sex (male *vs.* female)	329	0.93 (0.58~1.48)	0.75
Lymphovascular invasion (positive *vs.* negative)	237	0.455 (0.258~0.800)	0.06
Lymph node metastases (positive *vs.* negative)	276	0.58 (0.36~0.94)	0.03
Perineural invasion (positive *vs.* negative)	249	0.54 (0.32~0.89)	0.02
Stage (II *vs.* I)	73	0.13 (0.03~0.44)	2.88 × 10^−3^
Stage (IV *vs.* III)	226	0.5 (0.27~0.90)	0.02
Differentiation (moderate *vs.* well)	253	0.33 (0.17~0.62)	6.16 × 10^−4^
Differentiation (poor *vs.* moderate)	270	0.45 (0.23~0.84)	0.01
T stage (T2 *vs.* T1)	129	0.21 (0.08~053)	1.29 × 10^−3^
T stage (T3 *vs.* T1)	96	0.39 (0.14~0.99)	0.05
T stage (T4 *vs.* T1)	139	0.27 (0.10~0.65)	5.27 × 10^−3^

### Upregulation of CSTB Inhibits Malignant Biological Behaviors of OSCC Cell Lines

**Figure 3:**
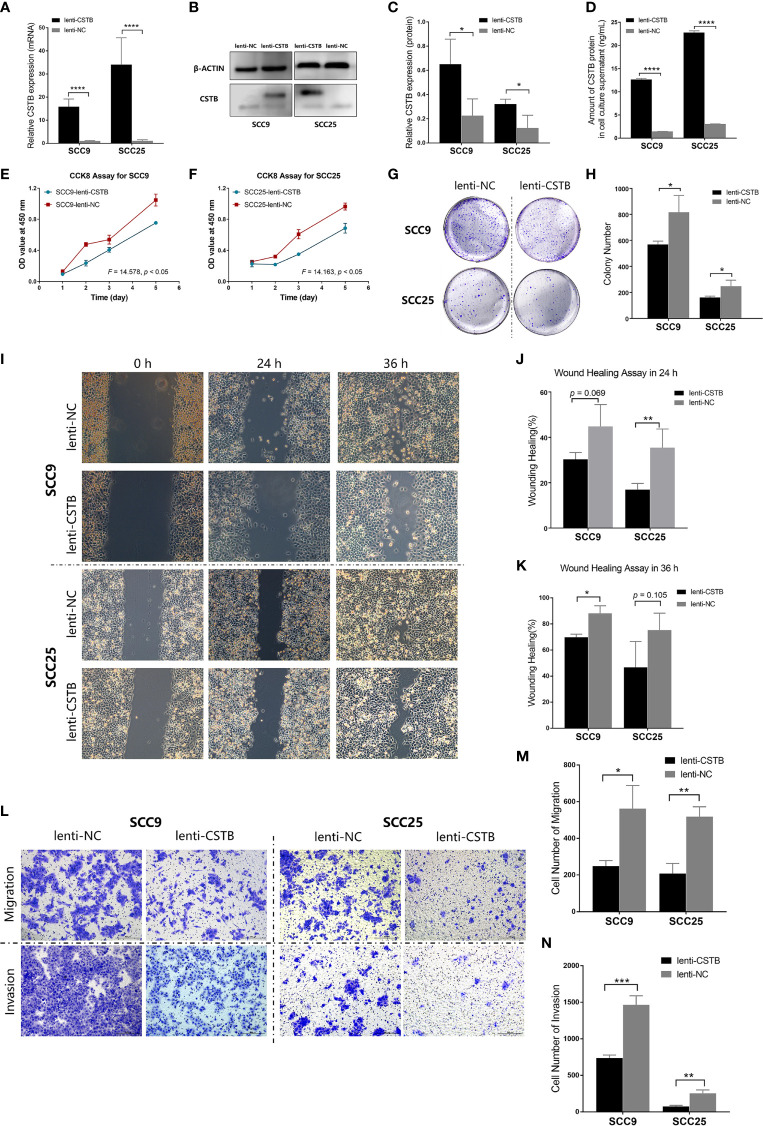
Upregulation of CSTB inhibits the proliferation, migration, invasion and adhesion of OSCC cell lines. **(A)** Relative expression of CSTB mRNA in OSCC cell lines after lentivirus transfection. **(B, C)** CSTB protein expression in OSCC cell lines after lentivirus transfection was identified by Western blot analysis **(B)** and quantitatively analyzed **(C)**. **(D)** The content of CSTB in the culture supernatant of OSCC cell lines after lentivirus transfection was quantified by ELISA. **(E, F)** CCK8 assays for transfected OSCC cell lines. **(G, H)** Colony formation assays for transfected OSCC cell lines. **(G)** The representative staining images of each group; **(H)** the number of clones formed in each group. **(I**–**K)** Wound-healing assays for transfected OSCC cell lines. Representative images in **(I)** and statistical results in **(J, K)** at 0, 24, and 36 h in each group. **(L**–**N)** Transwell assays for transfected OSCC cell lines. Representative images for cell migration and invasion of each group are shown in **(L)**. Statistical results for cell migration and invasion are shown in **(M, N)**, respectively. Error bars represent the standard deviation. *p < 0.05, **p < 0.01, ***p < 0.001, ****p < 0.0001.

Next, the regulatory role of CSTB in the malignant behaviors of OSCC was explored *in vitro*. CSTB was successfully transfected into OSCC cell lines (SCC9 and SCC25), and its content was upregulated both intracellularly and extracellularly (**Figures 3A–D**). Overexpression of CSTB suppressed the proliferation of SCC9 and SCC25 cells (**Figures 3E, F**) and caused a reduction in colony formation by 17% to 18% (**Figures 3G, H**). Compared with the NC groups, the wound-healing rate was decreased by 5% to 31% in SCC9/25 cells (**Figures 3I–K**). Regarding the invasive characteristics, overexpression of CSTB reduced the migration ability by ~37% and the invasion ability by 23%–38% in OSCC cell lines (**Figures 3L–N**).

### WGCNA to Identify the Target Module

**Figure 4:**
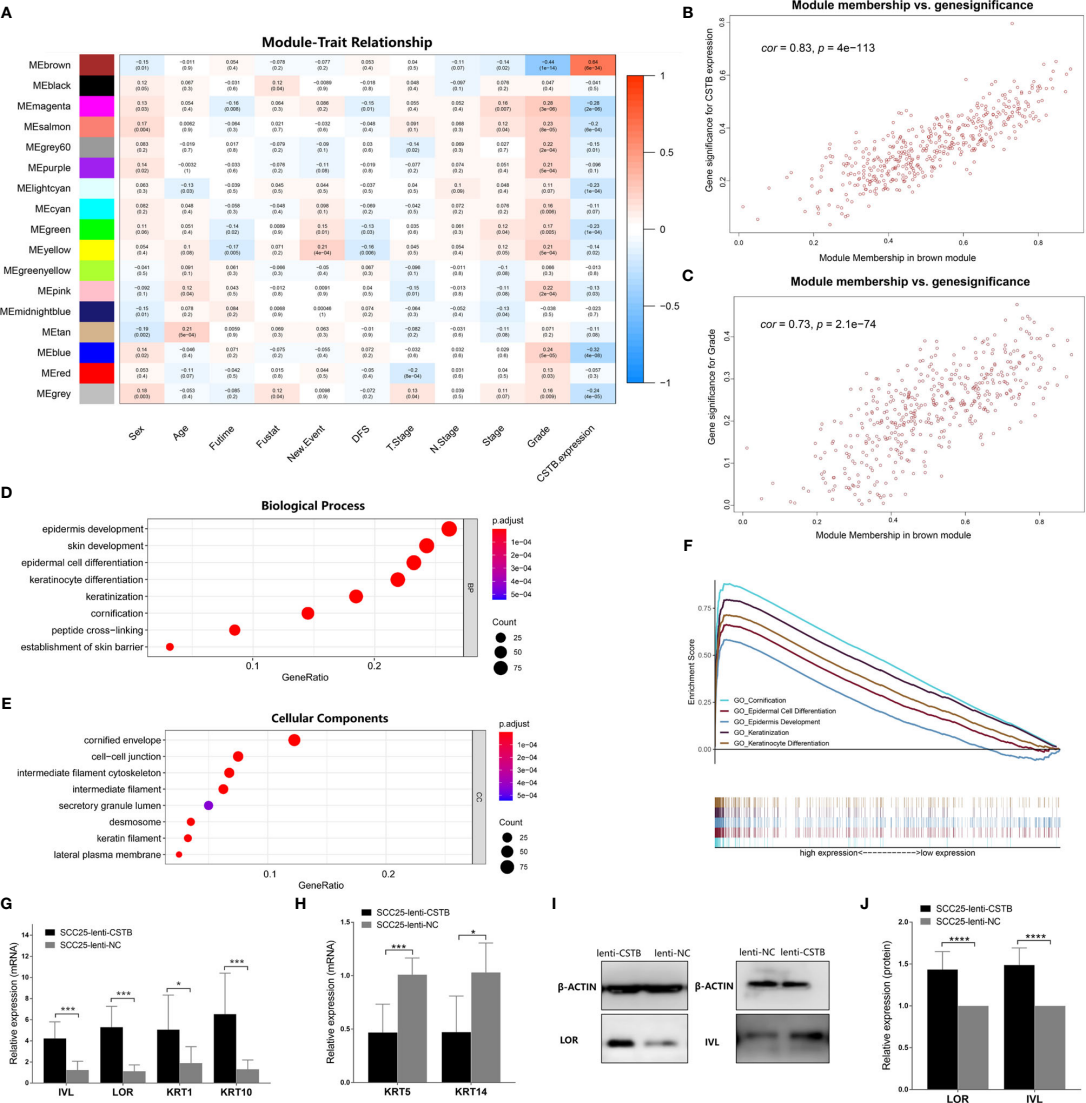
CSTB may regulate epithelial cell differentiation and keratinization in OSCC. **(A)** Heatmap of the correlation between module eigengenes (vertical axis) and clinical features (horizontal axis). The correlation coefficient (upper row) and *p*-value (lower row) are presented in each block. The color of each block represents the correlation coefficient according to the legend axis on the right. **(B)** Scatter diagram for the correlation between module membership and the gene significance of CSTB expression in the brown module. *Cor*, correlation coefficient; *p*, *p*-value. **(C)** Scatter diagram for the correlation between module membership and gene significance of grade classification in the brown module. *Cor*, correlation coefficient; *p*, *p*-value. **(D, E)** GO enrichment analysis of the brown module genes. **(F)** Squamous epithelial keratinization- and differentiation-related pathways with statistically significant differences in GSEA using KEGG enrichment analysis. **(G, H)** Relative mRNA expression of cell differentiation and keratinization-related markers in OSCC cell lines after lentivirus transfection. **(I, J)** Protein expression of cell differentiation and keratinization-related markers in OSCC cell lines after lentivirus transfection. Error bars represent the standard deviation. *p < 0.05, **p < 0.01, ***p < 0.001, ****p <  0.0001.

To explore the relevant mechanism of CSTB in OSCC, WGCNA was used as a nontargeted method to identify gene sets that were highly synergistic with CSTB. In WGCNA, the top 25% variant PCGs (a total of 5,716 genes) were selected as the input in the analysis. Thirteen outlier samples were removed, and the remaining 278 samples were clustered with clinical features and *Cstb* expression (**Figure S1**). A soft threshold power of *β* = 6 was selected to establish a ToM and further construct a scale-free network (**Figure S2**). The clustering tree was divided into 18 modules using dynamic shearing, and the modules were merged according to the coefficient of dissimilarity of <0.25 (**Figure S3**). The relationships between the input features and the module eigengenes are shown in **Figure 4A**. Interestingly, the module most associated with *Cstb* expression (i.e., the brown module, *r* = 0.83, *p* = 4 × 10^−113^, **Figures 4A, B** **)** was also the one most associated with grade classification (*r* = 0.73, *p* = 2.1 × 10^−74^, **Figures 4A, C** **)**. This result suggests that the gene set highly coexpressed with *Cstb* may have a certain effect on the grade classification phenotype, which indicated that CSTB may play a regulatory role in the phenotype of grade classification.

### CSTB Regulates Neoplastic Epithelial Differentiation and Keratinization in OSCC

Given the strong association of the brown module with the two traits (i.e., CSTB expression and grade classification), it was regarded as the target module for further GO enrichment analysis (**Supplementary Data Sheet 2**). The top 10 enriched biological processes (BPs) mainly included pathways associated with the epithelial proliferation/differentiation program, such as epidermal development, epidermal cell differentiation, and keratinocyte differentiation (**Figure 4D**). The top 10 enriched cellular components (CCs) consisted of cellular structures associated with epithelial proliferation/differentiation, including cornified envelopes, intermediate filament cytoskeleton, intermediate filament, etc. (**Figure 4E**). Moreover, GSEA was conducted as a targeted method to verify whether the pathways related to epithelial differentiation and keratinization showed differences between the high and low *Cstb* expression groups. The results showed that the high *Cstb* expression group activated more epithelial differentiation and keratinization processes (e.g., cornification, epidermal cell differentiation epidemics development, etc.) (**Figure 4F**).

Finally, cell differentiation/keratinization-related markers (i.e., IVL, LOR, KRT1, KRT5, KRT10, and KRT14) were validated at the protein and gene levels in OSCC models overexpressing CSTB (**Figures 4G–J**). It was found that upregulation of CSTB promoted the expression of these markers, reflecting a tendency for differentiation and keratinization. In summary, CSTB might regulate the epithelial proliferation/differentiation program in OSCC.

### RNA Sequencing Derived Pathways/Genes of Interest Associated With CSTB in OSCC

**Figure 5:**
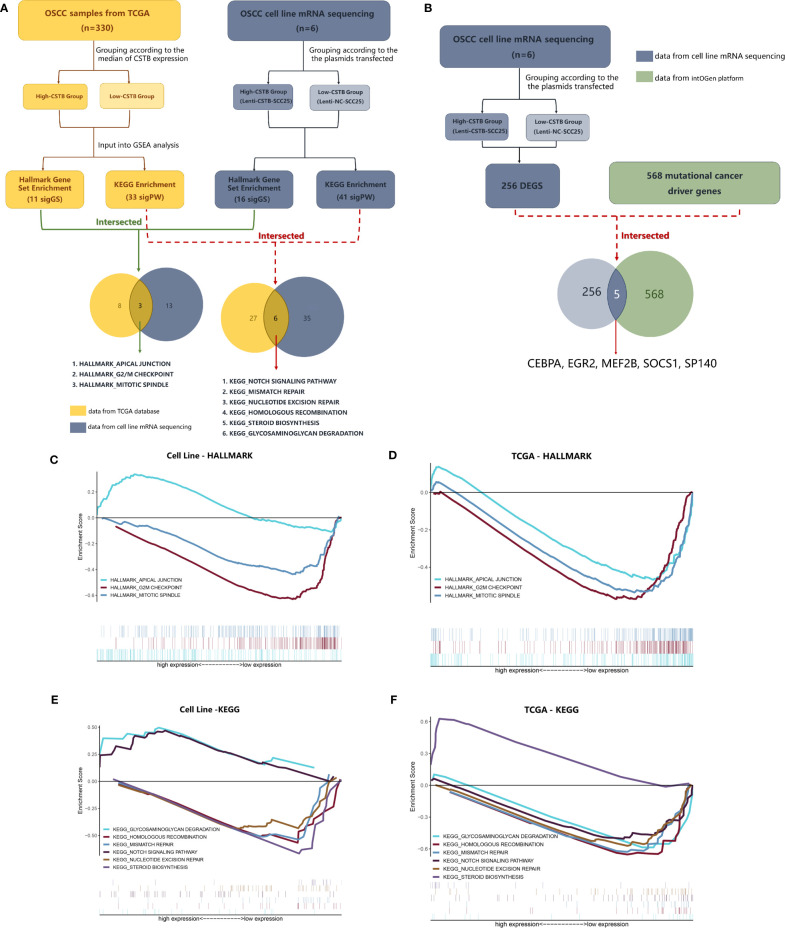
CSTB may participate in OSCC through other tumor-related pathways. **(A)** The process and results of GSEA based on data from the TCGA database/cell lines mRNA sequencing. **(B)** Comparison between mutational cancer driver genes and DEGs obtained from cell line mRNA sequencing. **(C, D)** GSEA using Hallmark gene sets. Gene sets with statistically significant differences based on data from the TCGA database/cell line mRNA sequencing. **(E, F)** GSEA using KEGG enrichment analysis. Pathways with both statistically significant differences based on data from the TCGA database/cell line mRNA sequencing.

In addition to keratinization- and differentiation-related pathways, other pathways of interest that CSTB may participate in were also identified. A comparison was made between the GSEA enrichment results of RNA-seq data (from the OSCC models overexpressing CSTB) and existing public database data (**Supplementary Data Sheets 3–4**). Three gene sets (i.e., apical junction, G2/M checkpoint and mitotic spindle) and six pathways (i.e., glycosaminoglycan degradation, homologous recombination, mismatch repair, NOTCH signaling pathway, nucleotide excision repair, and steroid biosynthesis) were enriched in the data from both sources (**Figures 5A, C–F**). These nine pathways/gene sets were named as shared pathways/gene sets. In the GSEA results for the cell line RNA-seq data, the core enrichment genes in the above shared pathways/gene sets were used to compare with 256 DEGs in order to obtain crossover genes (**Table 3**, No. 1–12), suggesting that overexpression of CSTB may be involved in the above shared pathways by regulating these 12 crossover genes.

**Table 3:** Highlighted genes out of 256 differentially expressed genes.

Items	No.	Gene	Fold change	*p-*Value	Enriched pathway/gene sets	Description in NCBI	Reported in HNSCC
Enriched in KEGG pathways/Hallmark gene sets in GSEA	1	MAML3	1.84	<0.01	Notch signaling pathway	Mastermind-like transcriptional coactivator 3	–
	2	NOTCH3	1.78	<0.01		notch receptor 3	(39–41)
	3	HSD17B7	−1.55	0.01	Steroid biosynthesis	Hydroxysteroid 17-beta dehydrogenase 7	(42)
	4	HS3ST3B1	1.80	<0.01	Glycosaminoglycan degradation	Heparan sulfate-glucosamine 3-sulfotransferase 3B1	–
	5	IDUA	1.64	0.03		alpha-l-iduronidase	/
	6	CRB3	1.79	<0.01	apical junction	crumbs cell polarity complex component 3	(43)
	7	CX3CL1	1.73	<0.01		C-X3-C motif chemokine ligand 1	(44–46)
	8	GRB7	1.67	<0.01		growth factor receptor bound protein 7	(47)
	9	ACTN2	3.14	<0.01		actinin alpha 2	(48, 49)
	10	MYBL2	−1.84	<0.01	G2/M checkpoint	MYB proto-oncogene like 2	(50)
	11	PIF1	−1.51	0.05	mitotic spindle	PIF1 5′-to-3′ DNA helicase	–
	12	DOCK2	−1.61	<0.01		dedicator of cytokinesis 2	–
Mutated cancer driver genes	13	CEBPA	−1.69	0.02	–	CCAAT enhancer binding protein alpha	(51, 52)
	14	EGR2	1.59	0.01	–	early growth response 2	(53, 54)
	15	MEF2B	1.51	0.01	–	Myocyte enhancer factor 2B	–
	16	SOCS1	−1.62	<0.01	–	Suppressor of cytokine signaling 1	(55, 56)
	17	SP140	−1.49	<0.01	–	SP140 nuclear body protein	(57, 58)

Genes No. 1–12 are the crossover genes obtained by comparing 256 DEGs with the core enrichment genes in the shared pathways/gene sets. Genes No. 13–17 are identified as the existing recognized mutational cancer driver genes in 256 DEGs. In the last column (“Reported in HNSCC”), “–” means that the associations of these genes with HNSCC have not been reported in the literatures and may be innovative findings.

In addition to exploration at the pathway level, RNA-seq data were used for comparisons with the existing recognized mutational cancer driver genes. Among the 256 DEGs, five genes (i.e., CEBPA, EGR2, MEF2B, SOCS1, and SP140) were recognized as mutated cancer driver genes (**Figure 5B**). Overexpression of CSTB can upregulate EGR2 and MEF2B while downregulating CEBPA, SOCS1, and SP140 in OSCC cell lines (**Table 3**, No. 13–17).

## Discussion

The roles of CSTB, a cysteine cathepsin inhibitor associated with various cancers, have been poorly understood, and few studies have focused on its association with HNSCC. The present study focused on OSCC and explored the regulatory roles of CSTB *in vivo* and *in vitro* with both experimental and bioinformatic data. The findings supported our hypothesis and showed that CSTB was downregulated in OSCC compared with normal tissues, and this downregulation was related to worse clinical outcomes or signs of tumor malignancy. The promoting role of CSTB in the OSCC proliferation-differentiation program was unprecedentedly proposed. In addition, some relevant mechanisms by which CSTB might participate in OSCC were suggested at both the pathway and gene levels.

In OSCC, we found that CSTB was downregulated both *in vivo* and *in vitro*, both at the protein and gene levels. CSTB has been reported to have abnormal expression in various types of tumors, in which the variation trend of CSTB was inconsistent between different tumors. For instance, CSTB seems to be upregulated in HCC (25–28), epithelial ovarian tumors (29, 30), and breast cancer (31), but downregulated in ESCC (32, 59) and laryngeal squamous cell carcinoma (33) (another certain kind of HNSCC). To systematically compare the expression of CSTB in common tumor types, the GEPIA database (60) was used to show the difference in CSTB expression between the tumor and normal groups (**Figure S4**): (1). CSTB was downregulated in HNSCC, which was consistent with the findings of our study and a previous study on HNSCC (i.e., laryngeal squamous cell carcinoma) (33) (2). CSTB was upregulated in most carcinomas (i.e., malignant tumors of epithelial origin), except for HNSCC and ESCC. This radically different trend of expression may be partly explained by the different histological origins of these epithelial-derived tumors. Among these carcinomas, HNSCC and ESCC share a common histological origin (i.e., stratified squamous epithelium), while other carcinomas originate from simple columnar epithelium (e.g., cholangiocarcinoma, colon adenocarcinoma, cervical squamous cell carcinoma, and uterine corpus endometrial carcinoma) or glandular epithelium (e.g., pancreatic adenocarcinoma, rectum adenocarcinoma, and stomach adenocarcinoma). In addition, the pleiotropic roles of CSTB in tumors may also contribute to this inconsistent trend. For instance, knockdown of CSTB in an epithelial ovarian cancer cell line inhibited cell proliferation and promoted apoptosis (29), whereas the opposite result was found in a gastric cancer cell line (61) (3). The same expression trend of CSTB in HNSCC and ESCC could be attributed to the similarity of these two tumors. First, they are both located in the proximal digestive tract and originate from stratified squamous epithelium, sharing some similar histological characteristics under physiological and pathological (i.e., appearance of keratin pearls in tumor status) conditions (62). Second, the histological distributions of CSTB in HNSCC and ESCC are also consistent, that is, it is located in the epithelial structure of carcinoma tissues (59) (**Figure 2I**). Under physiological conditions, CSTB is located in the epithelial layer of both normal oral cavity tissues and esophageal tissues (**Figures 2I** and **S5**). Third, similarities in genomic characterization between ESCC and HNSCC may also lead to the same expression trend of CSTB (7, 63–67). There are some groups of gene sets that mutate in both ESCC and HNSCC, which indicates that common mechanisms can regulate the initiation of squamous cell carcinoma (SCC) across different tissues (7, 8, 62, 64, 68–72).

As the expression trend of CSTB differs from tumor to tumor, the relationship between CSTB and prognosis is also inconsistent in different tumor types. Elevated levels of CSTB in tumor samples were related to a higher risk of recurrence and advanced tumor stages in bladder cancer (34) and were linked to a higher risk of tumor metastasis in HCC (27). In our study on OSCC, CSTB expression was inversely related to the risk of aggressive tumor characteristics (i.e., lymph node metastasis, perineural infiltration, and lymphovascular invasion). These characteristics favor tumor recurrence and metastasis (73) and pose great challenges to the tumor-free goal of radical surgery (73–79). Enhanced cancer cell migration and the capacity of stromal infiltration are essential steps of OSCC invasion and metastasis, as is the proliferation of cancer cells in metastatic foci (80). These three abilities of OSCC cells were regulated by CSTB *in vitro* in the present experiments. Upregulation of CSTB inhibited the proliferation of OSCC cells, regardless of whether proliferation was initiated by a group of cells (i.e., CCK-8 assays) or by a single cell (i.e., colony formation assays). The inhibitory effect of CSTB on cell migration and invasion seems to be more obvious, whether it was simple cell migration movement or invasion requiring the degradation of ECM. These results might partly explain the lower DFS rate of OSCC patients in the low CSTB expression group, given that DFS is an indicator of tumor recurrence and metastasis. In addition, in the early stages of OSCC (i.e., lower clinical stage or pathological grade), the expression changes of CSTB seemed to be more obvious, and the correlation with the corresponding clinical features (stages I–II or grades 1–2) was higher. This result indicated that CSTB might have more dramatic changes in the early stage of disease and might be better at distinguishing and predicting early OSCC. CSTB had no obvious relationship with demographic characteristics, including age and sex, suggesting a relatively stable expression of CSTB in the physiological state. Instead, CSTB tends to be changed specifically under pathological conditions [e.g., cancer (21), viral infection (81–83), and neurodegenerative diseases (84, 85)], which suggests that it is a potential pathological marker. The relationship between CSTB and the prognosis of OSCC in our study was also consistent with previous research reporting that low expression of CSTB in neoplastic islands from the invasive tumor front (ITF) was related to local recurrence in OSCC (35). Other studies of HNSCC/ESCC also reported that a lower concentration of CSTB increased the risks of lymph node metastasis (35, 59) and local tumor recurrence (35) and may thus result in a shorter DFS (35, 86). The different expression patterns (i.e., different expression trends and inconsistent relationships with prognosis) of CSTB in tumors imply that CSTB could be a tumor-specific whistleblower. For instance, when tumoral diseases are speculated to have tissular or humoral changes in CSTB, the types or primary focus of tumors could be predicted according to the change modes of CSTB.

Another major finding was that CSTB was involved in the squamous epithelium proliferation-differentiation program. This finding was supported by several lines of evidence, as follows (1). In WGCNA, the gene modules most associated with CSTB were also closely related to epithelial development, differentiation and keratinization (2). In GSEA, epithelial differentiation and keratinization processes were more active in the high CSTB expression group (3). The expression of CSTB was positively related to the degree of tumor differentiation at the protein level (IHC). In WGCNA based on TCGA data, CSTB and tumor grade classification showed an indirect relationship at the gene level through the bridging role of the brown gene module; that is, the gene module most associated with CSTB expression was also the one most closely related to the phenotype of grade (4). Upregulation of CSTB increased epithelial differentiation/keratinization markers [i.e., IVL (87, 88), LOR (87, 89), and KRT1/10 (90, 91)] and decreased epithelial basal-like markers [i.e., KRT5/14 (90, 91)]. While our study provided a direct evidence on the association between CSTB and squamous epithelium differentiation, previous studies on other diseases have implied a possible link between CSTB and epithelial differentiation. In ESCC, CSTB always exists in the clearly differentiated cells rather than basal-like cells, and the expression of CSTB disappears after malignant transformation of keratinocytes (92). Moreover, in psoriasis, a condition that manifests as uncontrolled keratinocyte proliferation and epithelial hyperplasia (93, 94), the amount of CSTB is increased, which is usually not seen in healthy epidermis (92) (i.e., normal skin). The regulatory mechanisms of CSTB in squamous epithelial keratinization and differentiation programs are not yet clear. There might be at least two possible mechanisms, i.e., by affecting the formation of cornified cell envelopes (CE) or by regulating the NOTCH signaling pathway. First, the terminal differentiation of squamous epithelium is a process accompanied by the formation of CE (95), which is mediated and regulated by TGM1 [transglutaminase-1 (TGase-1)] (96, 97). In our study, the CE was enriched as a cell component in GO analysis in the brown module in WGCNA (**Figure 4E**) and TGM1 was a significant hub gene in the brown module (module membership = 0.85, module eigengene = 0.65). Given that the brown module was a gene module containing genes coexpressed with CSTB, these results suggested that CSTB might regulate the TGM1-mediated CE formation process and ultimately regulate the terminal differentiation of squamous epithelium. Second, NOTCH is mostly regarded as a tumor suppressor in HNSCC, and loss-of-function mutations in NOTCH1 are common events in HNSCC. The NOTCH signaling pathway plays an important role in the normal functioning of squamous epithelium development and differentiation (7, 62, 67, 98–100), acting as a promoter of keratinocyte differentiation (101, 102). Conditional deletion of NOTCH1 in the mouse epidermis can lead to basal hyperplasia and basal cell carcinoma (103). Our GSEA results based on cell line RNA-seq data were consistent with this downregulation trend of NOTCH signaling. The NOTCH signaling pathway became more active after CSTB overexpression, which was also in line with the differentiation-promoting effect of CSTB that we have observed. However, this alteration in the NOTCH signaling pathway showed an opposite trend (i.e., more active in the low CSTB expression group) based on patient RNA-seq data from TCGA. This could be explained by the contextual and bimodal role of NOTCH in cancers, including HNSCC (64, 67, 104–106). NOTCH can act as an oncogene and tumor suppressor gene in different cell populations within the same tumor (103), which was a possible reason for the difference in NOTCH signals in the data from two sources (TCGA data and cell line RNA seq data). The cell line RNA-seq data came from a single type of tumor cell (OSCC cell line), while the tissue sequencing data of TCGA were composed of a variety of cells including tumor cells, stromal cells, immune cells, etc. Another possible reason for the different NOTCH signals is that the involved NOTCH paralogues and ligands vary in different disease contexts, resulting in different biological outcomes (107). For instance, in GSEA, NOTCH1, and NOTCH3 were enriched in the high CSTB expression group based on cell line RNA-seq data, while NOTCH1 and NOTCH4 were enriched in the low CSTB expression group based on TCGA data (**Supplementary Data Sheet 4**). Studies have identified a bimodal role for NOTCH3 in HNSCC (39), while MAML3 is essential for the operation of NOTCH signaling (108). In the RNA-seq data, we found that overexpression of CSTB could upregulate the expression of NOTCH3 and MAML3 *in vitro* (**Table 3**), suggesting that CSTB may be involved in the NOTCH signaling pathway by regulating these two genes, but additional validation is required.

An interesting phenomenon was found in our research: the content of extracellular/intracellular CSTB showed a consistent change trend in the *in vitro* experiment (**Figures 2M, D** **)**. CSTB is regarded as a protease inhibitor that is localized in the intracellular region (18, 23, 24). However, many studies have shown that CSTB and its changes can be detected in the body fluids of cancer patients [e.g., serum (28), ascites (109), and urine (34)] and are related to the clinical characteristics or prognosis. Given the consistency of the anatomical location, saliva as a body fluid may be more representative for reflecting the disease state of OSCC. A study of OSCC emphasized the potential of CSTB-specific peptides in saliva to reflect the status of lymph node metastasis in tumor-bearing patients (35). Although our study found differences in the content of CSTB in normal/OSCC cell culture supernatants, whether CSTB could be used to specifically identify tumor patients from the normal population is worthy of further research. Furthermore, a study showed the presence of CSTB in oral acquired enamel pellicles (110, 111). Future research may consider the changes in CSTB in plaque as a novel research direction in terms of the potential of CSTB as a biomarker, given that plaque could be regarded as a more stable form reflecting the biochemical composition of saliva. Other nontumor studies have identified certain extracellular effects of CSTB. CSTB in mouse synaptosomes can be secreted into cerebral spinal fluid in a depolarization-controlled manner and is involved in synaptic plasticity (84, 112). HIV-infected microglia secret more CSTB, participating in the neurotoxicity induced by cathepsin B (113). These studies suggest that pathological signals (tumor, virus, and abnormal nerve signals) may cause CSTB to translocate and participate in extracellular biological processes, while its specific extracellular role in tumors needs to be clarified by more tracer studies.

Other roles of CSTB in the context of OSCC were explored at both the pathway and gene levels in our study, which might provide a comprehensive perspective for further elucidating its mechanism. First, pathways related to the DNA damage repair mechanism (G2/M checkpoint, mitotic spindle, homologous recombination, mismatch repair, and nucleotide excision repair) were more active in the low CSTB expression group. This finding indicated that downregulation of CSTB might act as a predisposing factor of DNA damage in OSCC, thereby activating the pathways related to DNA damage repair pathways. Many studies have emphasized the protective effect of CSTB against oxidative stress (31, 114, 115). CSTB deficiency increases the sensitivity of cells to oxidative stress in cerebellar granule neurons (114) or breast cancer primary cells (31). Given that oxidative stress is a common factor that causes DNA damage (116, 117), we speculate that the effect of CSTB on the DNA damage repair mechanism in OSCC might be related to the loss of the protective role of CSTB in oxidative stress. Second, the identification of all the mutated genes capable of driving tumors is a landmark achievement towards tumor research (38). To explore the correlation between CSTB and the known cancer driver genes, we compared the DEGs obtained by cell line mRNA sequencing with the cancer driver gene set. As a result, five genes, CEBPA, EGR2, MEF2B, SOCS1, and SP140, were identified. CEBPA was reported to be mutated in HNSCC (51) and to play an important role in regulating the epidermal differentiation program (51, 52, 118–121). SP140 is an immune-related gene mutated in OSCC cell lines (57, 58, 122, 123). SOCS1, a negative regulator of cytokine signaling pathways (55, 124–126), was reported to regulate epigenetic modification in head and neck cancer (56, 125, 127). Cumulative studies have suggested the role of CSTB in immunomodulation, especially the relationship between CSTB and macrophages (128, 129). The lack of CSTB could transform macrophages to a proinflammatory phenotype by regulating related cytokines such as IL-10 (128). Meanwhile, some immune-related pathways (e.g., antigen processing and presentation and primary immunodeficiency) were enriched by GSEA based on cell RNA-seq data in our study (**Supplementary Data Sheet 3**). Both EGR2 and MEF2B are regulators of cell transcription (130, 131), and the former was reported to be involved in the epithelial-mesenchymal transition (EMT) pathway (131, 132), while the latter was differentially expressed between primary tumors and nodal metastasis tumor in HNSCC (53). Our experiments did not identify genes that are mutated due to changes in the expression level of CSTB, although changes in the expression level of the transcriptome are a common consequence of gene mutations. However, our aim was to interpret the possible role of CSTB in cancer at the genetic level, and the results still provide innovative insights while exploring the functions of CSTB in OSCC.

There were some limitations in our study. First, the correlations between *Cstb* and clinical characteristics found through data mining methods have not been confirmed by additional independent clinical data. The findings should be validated at the protein levels in tissues and body fluid (e.g., saliva and blood). Second, the regulatory role of CSTB in the epithelium differentiation phenotype was mainly recognized through bioinformatic analysis and *in vitro* experiments and needs *in vivo* confirmation in future studies. Third, the regulated pathways/genes identified by GSEA provided a relatively comprehensive perspective in clarifying the role of CSTB in OSCC. However, the relevant pathways/genes lacked targeted validation, so they should be interpreted with caution. Fourth, the sample size of OSCC tissue for IHC was relatively small, which was why we used Fisher’s exact test. In the subgroup analysis, the subgroup satisfying both high CSTB expression and moderate/poor differentiation included only one sample, which may be partly due to our relatively small overall sample size. Given the relationship of CSTB and differentiation in our study on OSCC, samples that meet both the phenotypes of high CSTB expression and low differentiation were relatively rare, which may be another reason for the small sample size of this subgroup. However, this state [i.e., high CSTB expression and poor tissue differentiation) is not uncommon in some other tumor types (e.g., HCC (27) and bladder cancer (34)], where the degree of tumor differentiation and CSTB expression show a negative correlation. This finding may further indicate that, compared with other tumor types, CSTB has a special additional role in the differentiation process of OSCC, but it is not the only factor determining the degree of tumor differentiation.

In conclusion, our study identified the modulatory role of CSTB in the malignant characteristics of OSCC for the first time and proposed some new relevant mechanisms, namely, CSTB may participate in OSCC by promoting the differentiation process of squamous epithelium. Our research provides a new perspective for interpreting the role of CSTB in tumors, especially in SCC. The specific role of CSTB in OSCC and its fine regulation of the squamous epithelial differentiation program still need to be explored in-depth by functional experiments and mechanistic research.

## Data Availability Statement

The original contributions presented in the study are included in the article/**Supplementary Material**. Further inquiries can be directed to the corresponding authors.

## Ethics Statement

Studies involving human participants were reviewed and approved by the Ethics Committee of the Stomatology Hospital of Guangzhou Medical University (LCYJ2021013). The patients/participants provided their written informed consent to participate in this study. No potentially identifiable human images or data are presented in this study.

## Author Contributions

TX contributed to the conception and design of the study. TX and LY performed the experiments. TX, XZ, XW, and LY performed the statistical analysis. TX wrote the first draft of the manuscript. TX, TY, and GL critically revised the manuscript. All authors contributed to the article and approved the submitted version.

## Funding

This study was supported by the Health Committee of Guangdong Province (Grant No. B2020209), Guangzhou Medical University (Grant No. C195015024), and the National Natural Science Foundation of China (Grant No. 81700985).

## Conflict of Interest

The authors declare that the research was conducted in the absence of any commercial or financial relationships that could be construed as a potential conflict of interest.

## Publisher’s Note

All claims expressed in this article are solely those of the authors and do not necessarily represent those of their affiliated organizations, or those of the publisher, the editors and the reviewers. Any product that may be evaluated in this article, or claim that may be made by its manufacturer, is not guaranteed or endorsed by the publisher.
