# Supplementary material for: Cystatin-B Negatively Regulates the Malignant Characteristics of Oral Squamous Cell Carcinoma Possibly Via the Epithelium Proliferation/Differentiation Program
Source: Front Oncol. 2021 Aug 24;11:707066. doi: 10.3389/fonc.2021.707066 (PMC8421684; doi:10.3389/fonc.2021.707066)
Supplement: Supplementary file 2 [file DataSheet_2.docx]

**S1** **Materials and methods**

**S1.1 Clinical patient specimens and immunohistochemistry (IHC) staining**

Immunohistochemical staining (IHC) was performed for 23 human OSCC tumor tissues and 15 normal mucosal epithelial tissues. Paraffin-embedded tissues were cut into 4 µm sections. They were dewaxed, dehydrated, and then were subjected to 3% H_2_O_2_ solution to inhibiting the activity of endogenous peroxidase. Antigen retrieval was carried by microwave heating with sodium citrate buffer (pH 7.8). Next, slides were blocked with 5% BSA solution. Tissue sections were incubated with primary antibody against CSTB (1:150 dilution, Zen-Bio) at 4 ℃ for 12 hours, and then were washed and incubated with second antibody (1:100 dilution, ZSJB-Bio) at 37℃ for 1 hour. Finally, slides were washed, incubated with diaminobenzidine (DAB, ZSJB-Bio), washed again and counterstained with hematoxylin.

**S1.2 Weighted Gene Co-expression Network Analysis (WGCNA)**

The gene expression data and samples clinical information were downloaded from TCGA. First, the variance analysis of 22862 PCGs were performed, the top 25%（5716 genes） of which were selected for WGCNA analysis. The top 25% variant genes and all the samples are examined by applying goodSamplesGenes function and sample clustering method respectively to rule out outlier genes and samples. The WGCNA package in R software was applied to construct a gene co-expression network. The soft threshold power (β = 6) was selected based on the scale independence and mean connectivity analysis to ensure a scale-free network. (Supplementary Data FigureS1B-D). The adjacency was calculated and an adjacency matrix was established. Afterwards, the adjacency matrix was transformed into a topological overlap matrix (ToM) and the corresponding dissimilarity was calculated. ToM was then used to construct a hierarchical clustering gene tree, and gene co-expression modules were identified using dynamic tree cut method. The module eigengene（ME）was defined as the first principal component of a specific module, representing the overall trend and level of gene expression within the module. The module eigengenes were calculated to identify modules that were significantly associated with the clinical feature information. In this process, expression level of CSTB was also regarded as a feature and was input into the correlation analysis, aiming to identify the functional modules related to its expression. Therefore, modules with a high correlation coefficient are considered candidate modules related to clinical features and selected for subsequent analysis. Finally, gene ontology (GO) and Kyoto Encyclopedia of Genes and Genomes (KEGG) enrichment analyses were performed to reveal functions of gene in the target modules.

**S1.3 mRNA Sequencing**

The total RNA of transfected SCC25 (lenti-CSTB-SCC25 and lenti-NC-SCC25) were extracted for further RNA sequencing analysis (n=3). High-throughput sequencing was conducted by SEQHEALTH (Wuhan, China). 2 μg total RNAs were used for stranded RNA sequencing library preparation using KCTM Stranded mRNA Library Prep Kit for Illumina® (Catalog NO. DR08402, Wuhan Seqhealth Co., Ltd. China) following the manufacturer’s instruction. PCR products corresponding to 200-500 bps were enriched, quantified and finally sequenced on Hiseq X 10 sequencer (Illumina) and raw reads of samples were obtained. Q30 is regarded as the standard of quality control, Q30>85% means that the sequencing quality is acceptable for further experiment. Trimmomatic software was used to remove joints as well as low quality reads and obtain high quality clean reads. Afterwards, the clean reads were mapped to the reference genome of Homo sapiens （Homo_sapiens.GRCh38; ftp://ftp.ensembl.org/pub/release-87/fasta/homo_sapiens/dna/） using STRA software (version 2.5.3a) with default parameters. Reads mapped to the exon regions of each gene were counted by featureCounts (Subread-1.5.1; Bioconductor) and then RPKMs were calculated.

Table S1
RT qPCR primers used in this study

| Genes | Forward Primers (5–3′) | Reverse Primers (5–3′) |
| --- | --- | --- |
| *GAPDH* | GGAGCGAGATCCCTCCAAAAT | GGCTGTTGTCATACTTCTCATGG |
| *CSTB* | AGGTCCCAGCTTGAAGAGAAA | CGCAGGTGTACGAAGTCCTC |
| *KRT1* | GTGATGCAGAGCAGCGT | GTGATGCAGAGCAGCGT |
| *KRT10* | GTGGTATGAAAAGCATGGC | AAGCAGGATGTTGGCATT |
| *KRT5* | CCTTCTGCGTCCTGCTG | GACTGGCGAGACATGGTG |
| *KRT14* | TCCTCCTCCCAGTTCTCCT | ACACCACCTTGCCATCG |
| *IVL* | GGTGGCCCGTCTCATCT | GTCATTGGGGTTGGCACT |
| *LOR* | CGTCTGCGGAGGTGGTT | TGCTTCTGCTGGGTCTGG |

Table S2

| GEO series | Contributor(s) | Tumor  (n) | Nontumor  (n) | Platform |
| --- | --- | --- | --- | --- |
| GSE30784 | [Chen C](https://www.ncbi.nlm.nih.gov/pubmed/?term=Chen%20C%5bAuthor%5d) et al | 167 | 45 | [HG-U133_Plus_2] Affymetrix Human Genome U133 Plus 2.0 Array |
| GSE42743 | [Holsinger C](https://www.ncbi.nlm.nih.gov/pubmed/?term=Holsinger%20C%5bAuthor%5d) et al | 74 | 29 | [HG-U133_Plus_2] Affymetrix Human Genome U133 Plus 2.0 Array |
| GSE25099 | [Peng C](https://www.ncbi.nlm.nih.gov/pubmed/?term=Peng%20C%5bAuthor%5d) et al | 57 | 22 | [HuEx-1_0-st] Affymetrix Human Exon 1.0 ST Array [transcript (gene) version] |
| GSE37991 | [Lee CH](https://www.ncbi.nlm.nih.gov/pubmed/?term=Lee%20CH%5bAuthor%5d) et al | 40 | 40 | Illumina HumanRef-8 v3.0 expression beadchip |
| GSE10121 | [Sticht C](https://www.ncbi.nlm.nih.gov/pubmed/?term=Sticht%20C%5bAuthor%5d) et al | 35 | 6 | DKFZ/Operon Human Oligo Set v4.0 |
| GSE85195 | [Bhosale PG](https://www.ncbi.nlm.nih.gov/pubmed/?term=Bhosale%20PG%5bAuthor%5d) et al | 34 | 15* | Agilent-014850 Whole Human Genome Microarray 4x44K G4112F |

* In GSE85195, samples in nontumor group were oral leukoplakia (OPL).

**Figures**

**Figures S1**


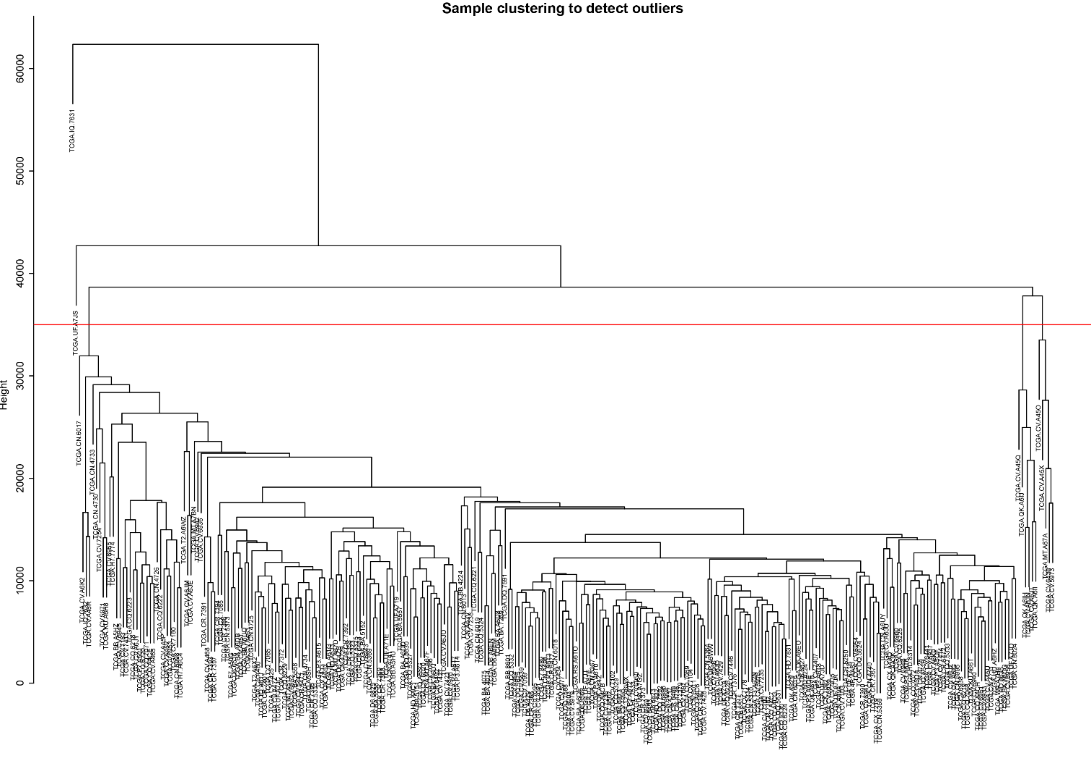


**FigureS1 Sample clustering to detect outliers in WGCNA**

13 outlier samples were removed and the remaining 278 samples were cluster

**Figure S2**


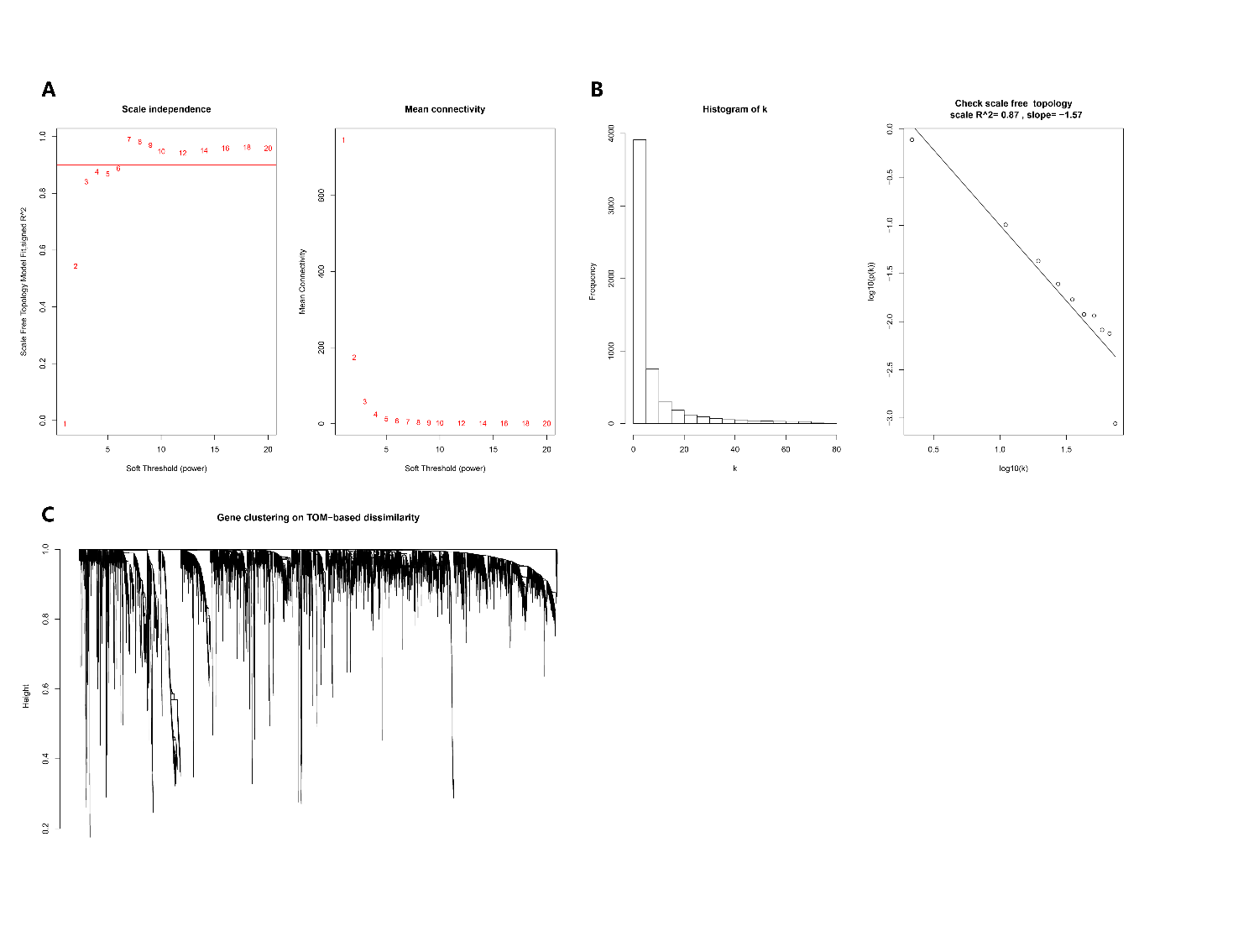


**Figure S2 The construction of a scale-free network.**

**(A)** Analysis of the scale independence and mean connectivity of different soft-thresholding powers; **(B)** Checking the scale free topology (*β* = 6) **（C）**The soft threshold power as β = 6 was selected to established a ToM and further constructed a scale-free network.

**Figure S3**


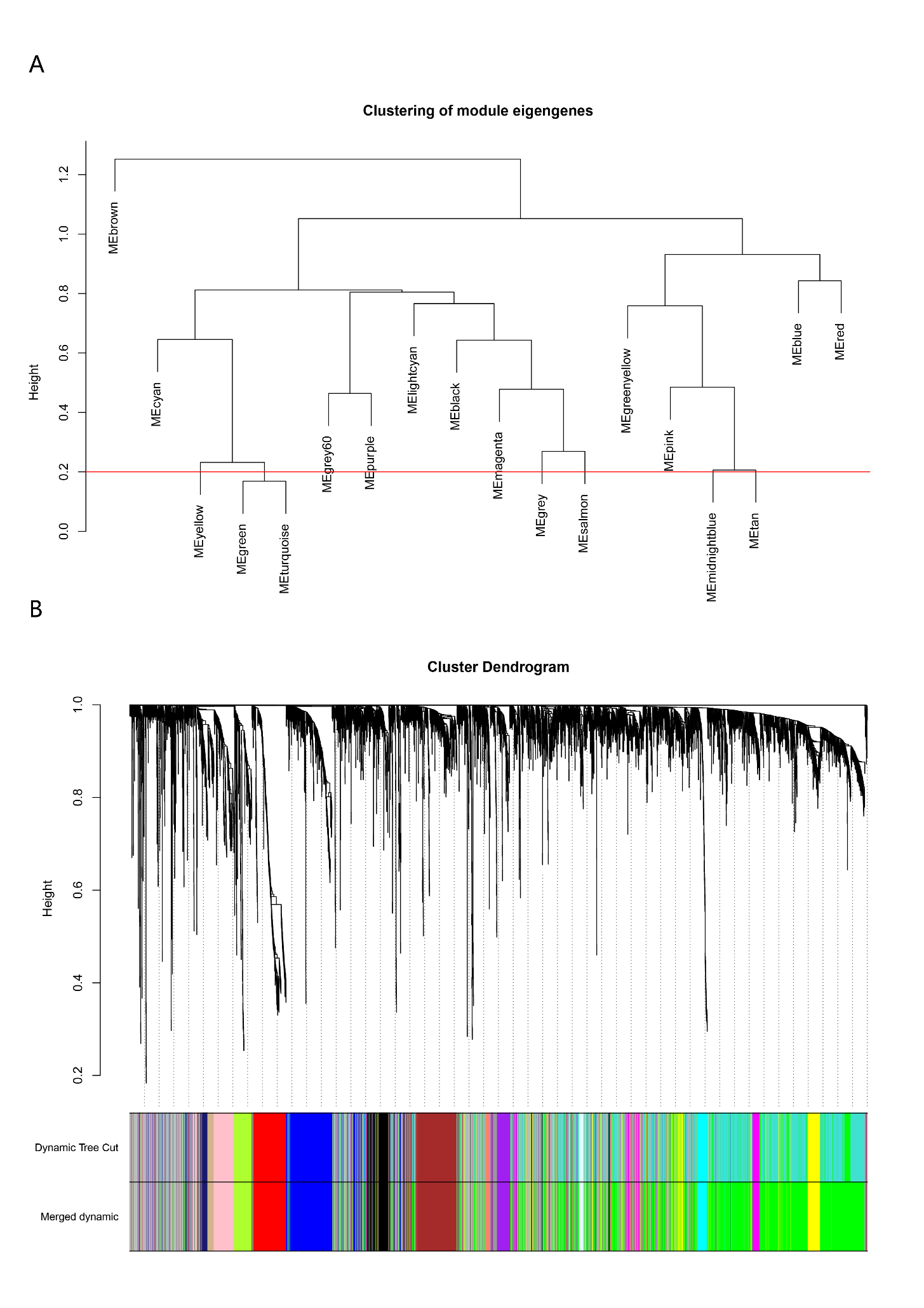


**Figure S3 Module visualization**

(A) The cluster dendrogram of module eigengenes. 18 gene modules were identifiled using using dynamic shearing (gray represents a gene not assigned to any module; the module’s minimum gene number is 30). The red line (height = 0.2) means the modules would merged according to the coefficient of dissimilarity of <0.2.

(B) Cluster dendrogram of genes in all samples. Each branch in the dendrogram represents one single gene. In the color panel, each color represent a gene module contaning co-expressed genes. The upper color panel (Dynamic tree cut) and the lower one (Merged dynamic) respectively represented the modules before and after merge.

**Figure S4**


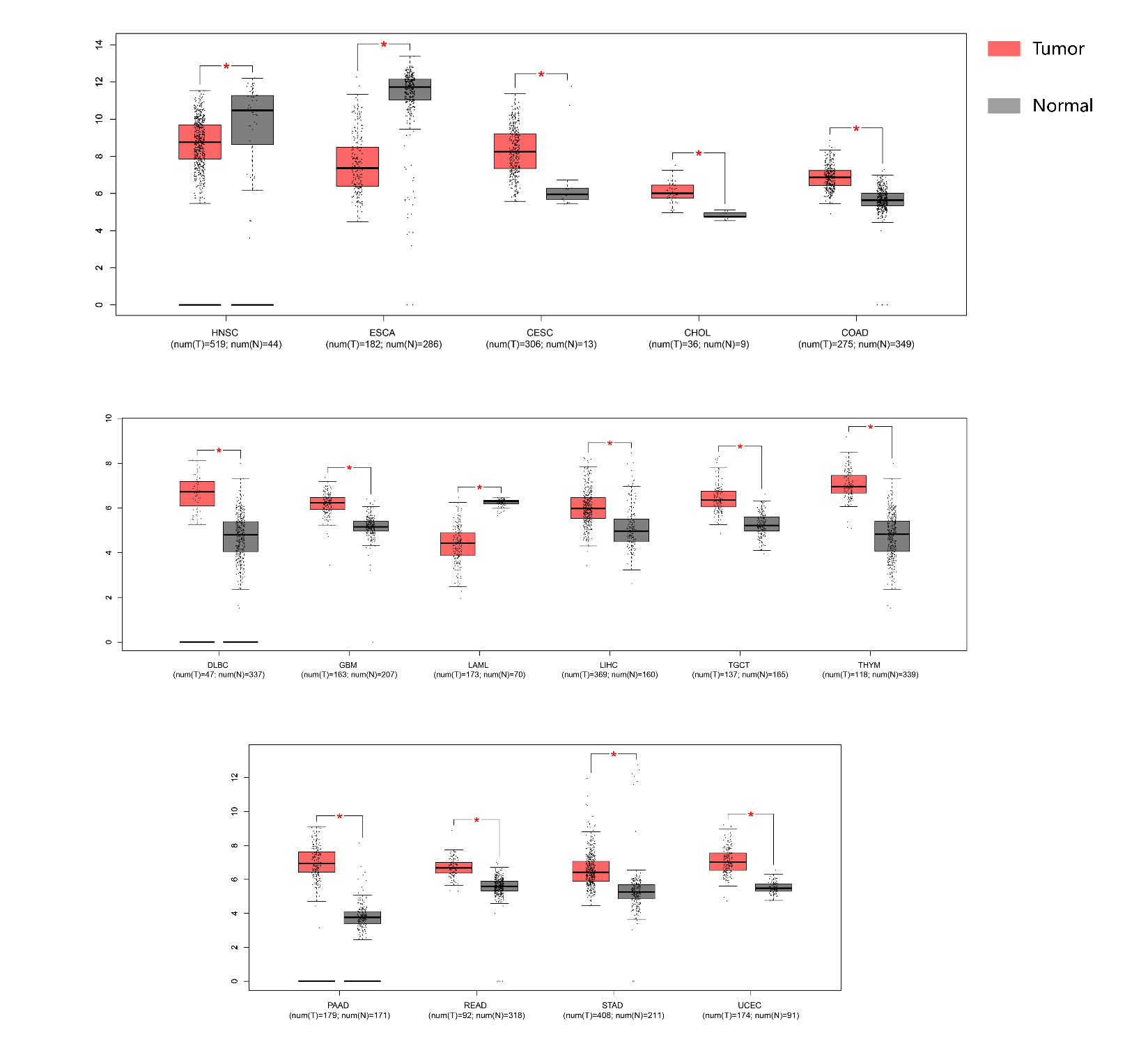


**Figure S4 The expression of CSTB in common tumor types**

Tumor types with differential expression of CSTB between tumor groups and normal groups (|Log2FC|>1; *p* < 0.05); Data from GEPIA (Gene Expression Profiling Interactive Analysis, http://gepia.cancer-pku.cn/ ); HNSC, Head And Neck Squamous Cell Carcinoma; ESCA, Esophageal Carcinoma; CESC, Cervical Squamous Cell Carcinoma; CHOL, Cholangiocarcinoma; COAD, Colon Adenocarcinoma; DLBC, Lymphoid Neoplasm Diffuse Large B-Cell Lymphoma; GBM, Glioblastoma Multiforme; LAML, Acute Myeloid Leukemia; LIHC, Liver Hepatocellular Carcinoma; TGCT, Testicular Germ Cell Tumors; THYM, Thymoma; PAAD, Pancreatic Adenocarcinoma; READ, Rectum Adenocarcinoma; STAD, Stomach Adenocarcinoma; UCEC, Uterine Corpus Endometrial Carcinoma.

**Figure S5**

**
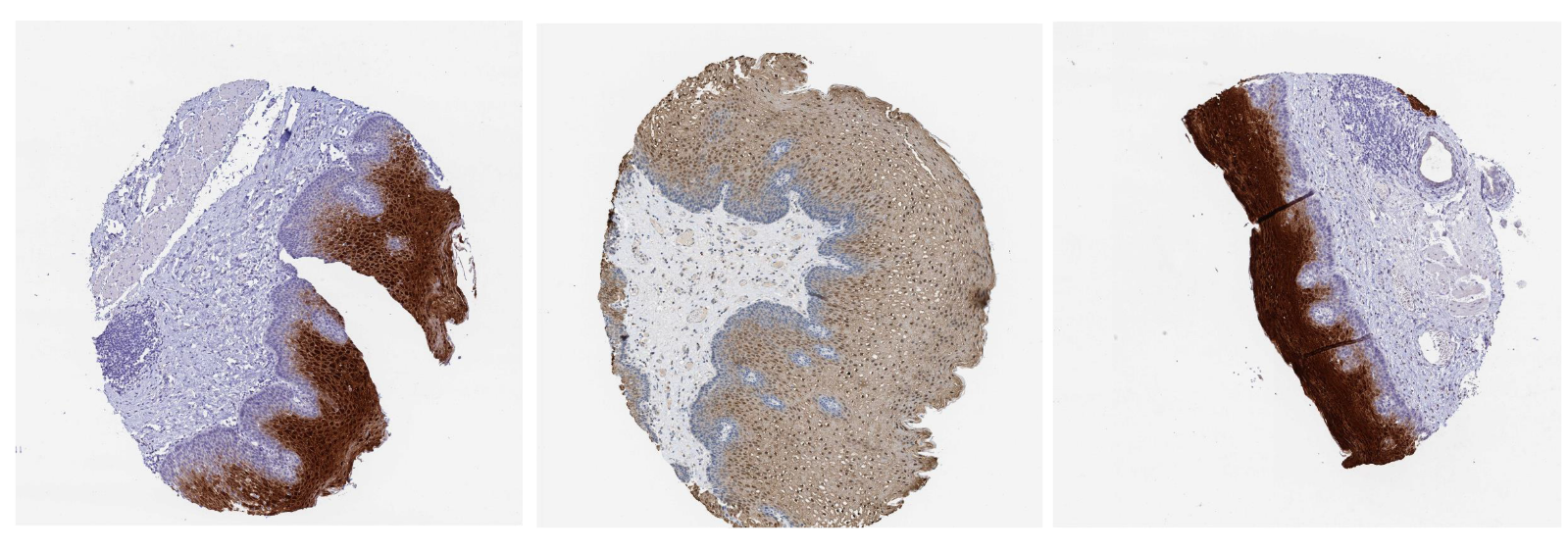
**

**Figure S5 CSTB expression in normal esophagus tissues.**

IHC staining of CSTB in normal esophagus tissues. Pictures from The Human Protein Atlas (https://www.proteinatlas.org/)
